# Physical Activity and Exertional Desaturation Are Associated with Mortality in Idiopathic Pulmonary Fibrosis

**DOI:** 10.3390/jcm5080073

**Published:** 2016-08-18

**Authors:** Baruch Vainshelboim, Mordechai Reuven Kramer, Shimon Izhakian, Ricardo M. Lima, Jose Oliveira

**Affiliations:** 1 Pulmonary Institute, Rabin Medical Center, Beilinson Hospital, Petach Tikva 4941492, Israel; kramerm@clalit.org.il (M.R.K.); shimixyz@gmail.com (S.I.); 2 Research Center in Physical Activity, Health and Leisure (CIAFEL), Faculty of Sport, University of Porto, Porto 4200-450, Portugal; joliveira@fade.up.pt; 3 Cardiology Division, Veterans Affairs Palo Alto Health Care System/Stanford University, Palo Alto, CA 94304, USA; professorricardomoreno@gmail.com; 4 Sackler Faculty of Medicine, Tel Aviv University, Tel Aviv 6997801, Israel; 5 Faculty of Physical Education, University of Brasília, Brasília 70910-900, Brazil

**Keywords:** international physical activity questionnaire, pulmonary rehabilitation, interstitial lung disease, exercise, inactivity

## Abstract

Idiopathic pulmonary fibrosis (IPF) is a chronic lung disease that manifests in hypoxemia, inactivity, and poor prognosis. This study aimed to assess the prognostic role of physical activity (PA) and exertional desaturation (ED) with mortality in IPF. At baseline, 34 IPF patients (68 (50–81) years) were interviewed using the International Physical Activity Questionnaire (IPAQ), and SpO_2_ was assessed pre to post 6-min walking test (∆SpO_2_). Patients were prospectively followed up for 40 months. Receiver operating characteristics curve analysis determined cut-off points associated with mortality, and Cox proportional hazard ratio (HR) were conducted. Thresholds for increased mortality risk in IPF patients were determined as IPAQ ≤ 417 metabolic equivalent task (METS)-min/week, *p* = 0.004 (HR; 9.7, CI 95% (1.3–71.9), *p* = 0.027), and ∆SpO_2_ ≥ 10%, *p* = 0.002, (HR; 23.3, CI 95% (1.5–365), *p* = 0.025). This study demonstrated a significant association of PA and ED with mortality in IPF patients. The findings emphasize the clinical importance of PA and ED assessments to aid in IPF risk stratification, prognosis prediction, and in providing early appropriate treatments, such as pulmonary rehabilitation, PA consultation, oxygen supplementation, and lung transplantation referral. These results underscore that even low levels of PA corresponding to 100–105 min/week were associated with a reduced mortality risk and better survival in IPF.

## 1. Introduction

Idiopathic pulmonary fibrosis (IPF) is a chronic, progressive interstitial lung disease (ILD) with substantial morbidity and mortality, poor prognosis, and an estimated median survival of 2 to 5 years from the time of diagnosis [1,2,3]. Exercise-induced dyspnea and desaturation are the hallmark characteristics of IPF patients; these are associated with disease severity and worse prognosis [1,4]. A threshold of SpO_2_ < 88% during 6-min walking test (6MWT) has been demonstrated to be associated with poor survival and is recommended by the American Thoracic Society (ATS)/European Respiratory Society (ERS) guidelines as a risk factor for mortality in IPF [1,4]. In addition, IPF patients presenting these signs and symptoms tend to be less physically active compared to age-matched healthy individuals, in order to avoid uncomfortable episodes, especially during exercise [3,5,6]. The importance of an active lifestyle was underscored by the World Health Organization in 2010, signifying physical inactivity as the 4th leading risk factor for global mortality [7]. Worldwide, 31% of adults are considered physically inactive, contributing to 6% of all deaths with an estimated 5.3 million premature deaths occurring in 2008 [7,8,9]. Moreover, among chronic respiratory disease patients, physical inactivity is associated with poorer outcomes, including higher mortality risk [10]. Nevertheless, physical activity behaviors were not studied extensively in patients with IPF; the few available reports show that inactivity is associated with more severe disease conditions and poorer survival [6,11,12].

In reviewing the literature addressing IPF, we detected some clinically important gaps related to levels of physical activity (PA) and exertional desaturation (ED) with mortality risk. Firstly, the previous proposed exertional nadir of absolute SpO_2_ < 88% may be different for patients with various resting saturation levels (with and without resting hypoxemia), and possibly has less prognostic sensitivity for all spectrums of disease severities. Secondly, physical inactivity is a well-established, independent risk factor for mortality in the general population [13] as well as in chronic obstructive pulmonary disease (COPD) patients [14]. Nonetheless, only limited data is available on IPF, and therefore it seems to be important to further explore the role of PA in this group of patients. Finally, IPF is considered as a fatal disease with poor prognosis and limited treatment options; good predictors of prognosis are still warranted for comprehensive risk stratification and for the implementation of effective interventions in the clinical setting. Considering the above-mentioned gaps in data, this study aimed to evaluate the prognostic role of PA assessed by the International Physical Activity Questionnaire (IPAQ) and exertional desaturation (measured by SpO_2_ pre- to post-6MWT (∆SpO_2_)) with mortality among patients with IPF.

## 2. Methods

### 2.1. Subjects and Setting

This observational prospective follow-up study was conducted at the Pulmonary Institute, Rabin Medical Center, Beilinson Hospital, Petach-Tikva, Israel. The study protocol was approved by the Ethics Committee of the hospital and was registered with clinicaltrials.gov (NCT01499745). The study was conducted in accordance with the Declaration of Helsinki and written informed consent was obtained from each subject prior to participation. Patients were included if they were diagnosed with IPF according to the accepted clinical-radiological criteria of the ATS/ERS and were clinically stable in previous 3–6 months [1]. Exclusion criteria were: severe co-morbid illnesses, unstable cardiac disease, and any neurological or orthopedic contraindications for exercise testing.

In the current study, subjects from our previous exercise training study [15] were followed up until 40 months from baseline. According to 50 m improvement in 6-min walking distance (6MWD), a power analysis prior to the recruitment revealed that a total of 30 participants were needed (15 in each group) to detect a significant difference between the groups [15,16]. Thirty-eight IPF subjects treated in the Pulmonary Institute were screened for eligibility, and 34 subjects volunteered and were recruited for participation in the study based on 10% expected dropouts reported in a previous ILD training study [17]. We have previously reported that at 30-months and at 40-months follow-up, group allocation did not affect survival in this group of subjects; however, the sample size was underpowered to detect such changes [18,19]. Demographic characteristics were obtained from all subjects and evaluation for dyspnea by the modified Medical Research Council (mMRC) scale was made [20,21]. In the current study, baseline data of subjects (*n* = 34) were used to determine the prognostic predictors of survival. Subjects were tested as described below and followed up for 40 months from baseline:

### 2.2. Physical Activity Assessment

Self-report 7-day short form International Physical Activity Questionnaire (IPAQ) was used for all patients [22,23]. Each patient was personally interviewed one-on-one, and each item in the questionnaire was explained to increase the accuracy of the self-reported PA. The questionnaire comprises nine items to assess the level of PA at moderate (4 metabolic equivalent task, METS) and vigorous intensities (8 METS), walking (3.3 METS), and sitting times. The score of overall PA was calculated in (MET)-min/week units, which is the sum of each mode of activity multiplied by the constant level of energy (MET) required for the task, as described above, by number of minutes performed per day, and by the amount of time performed per week [23,24]. Based on questionnaire’s manual, 600 METS-min/week (150 min/week * 4METS of moderate intensity PA) was considered a threshold for meeting the general PA recommendation [7,25,26,27,28]. 

### 2.3. Six-Minute Walking Test (6MWT)

The 6MWT was conducted according to ATS guidelines in a 35-meter corridor at the pulmonary unit within the hospital [29]. Borg dyspnea Category-Ratio (CR) 10 scale, heart rate and oxygen saturation (SpO_2_%; Pulse Oximeter 2500 30 EM, Nonim Medical, Minneapolis, MN, USA) were obtained prior and immediately after the test [26]. Oxygen supplementation during the test was provided for patients who were regular oxygen users at rest, while other patients received oxygen only after the test, as required. Exertional desaturation was defined as the difference in SpO_2_% pre- to post-6MWT (∆SpO_2_%).

### 2.4. Pulmonary Function Tests

Pulmonary function tests, including spirometry total lung capacity, maximal voluntary ventilation, and diffusion capacity for carbon monoxide were performed according to standard techniques and ATS/ERS guidelines (Zan 530 Oberthulba, Germany) [30,31,32]. All the measured parameters were presented as percentage of predicted (% predicted) values of the European Community for Coal and Steel [33].

### 2.5. Statistical Analysis

The statistical analyses were conducted using SPSS v.23 software (Chicago, IL, USA). The significance level was set at *p* < 0.05. Demographics, clinical, and physiological data of the participants are presented as median and (range). Categorical variables are presented in absolute values and (%) of group. Receiver operating characteristics (ROC) curve analysis for cut-off points’ ˃70% sensitivity and ˃70% specificity to detect mortality was performed for IPAQ and ∆SpO_2_. A multivariate Cox proportional hazard model using significant cut-off points determined from ROC was analyzed for IPAQ and ∆SpO_2_. The model was adjusted for gender, age, forced vital capacity %predicted, diffusion capacity for carbon monoxide %predicted, and Prednisone usage. Kaplan–Meir curves were used for survival analysis. Subjects who underwent lung transplants were considered as fatalities in the statistical analysis, as has been reported previously [34,35]. Exploratory data analysis using Spearman’s correlation coefficient was performed for PA levels (IPAQ score) and ED (∆SpO_2_) with 6 min walking distance (6MWD) [36]. PA and ED characteristics in survivor and non-survivor IPF patients were conducted using Chi Square test for categorical variables.

## 3. Results

The study population consisted of 34 IPF subjects from the Pulmonary Institute who were recruited for the pulmonary rehabilitation study [15]. All subjects completed the tests without adverse events. Demographics and general characteristics data are presented in Table 1. Nine percent of the patients were on Pirfenidone at baseline, while 24% used the drug during the follow-up. There were no other changes in medications during the study period.

**Figure 1:**
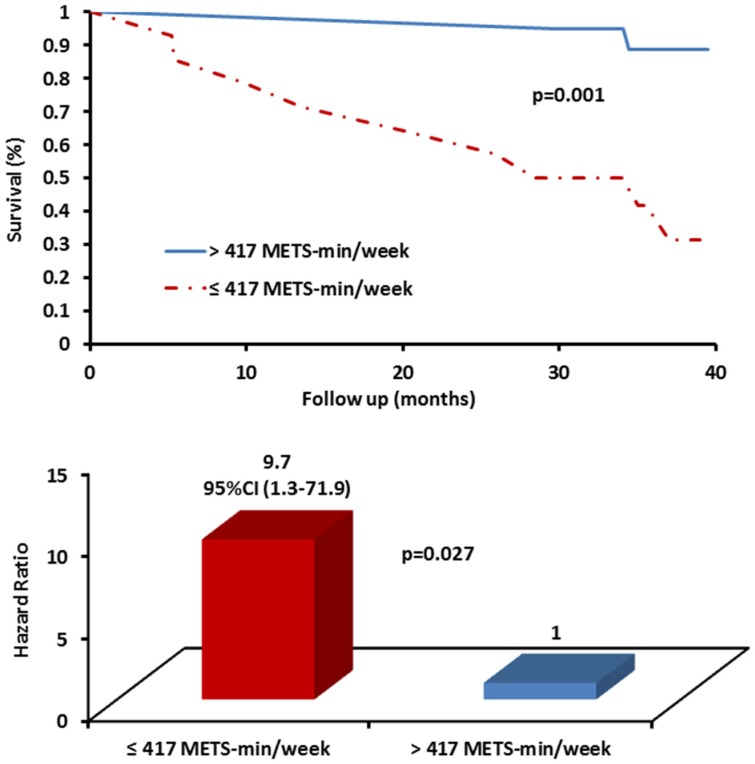
Kaplan–Meir survival curves and hazard ratio for physical activity threshold in patients with idiopathic pulmonary fibrosis.

**Figure 2:**
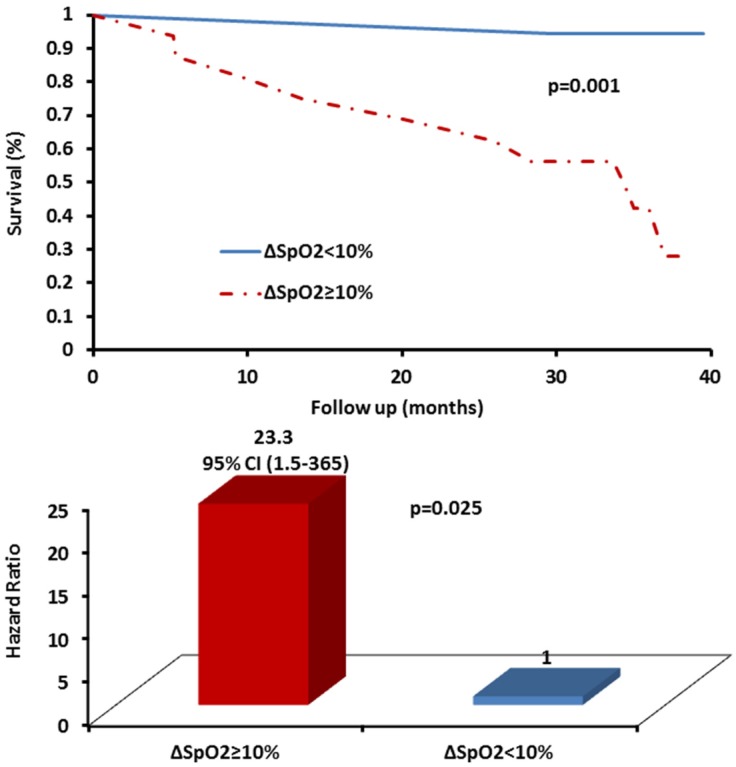
Kaplan–Meir survival curves and hazard ratio for exertional desaturation (∆SpO_2_) threshold in patients with idiopathic pulmonary fibrosis.

During the 40-month follow up, 11 subjects died (7 women and 4 men). Two of the 11 patients were at end-stage IPF and underwent lung transplants, and were considered as fatalities in the analysis. In the current study, significant cut-off points for IPAQ and ∆SpO_2_ were determined as prognostic predictors using ROC analysis (Table 2). Patients had poorer survival with significantly increased risk for mortality if they presented IPAQ ≤ 417 METS-min/week, *p* = 0.004, HR: 9.7, CI 95% (1.3–71.9), *p* = 0.027 and ∆SpO_2_ ≥ 10%, *p* = 0.002, HR; 23.3, CI 95% (1.5–365), *p* = 0.025. (Table 2; Figure 1 and Figure 2). Exploratory analysis showed significant correlation between PA and 6MWD (*r* = 0.57, *p* < 0.001) but not for ED and 6MWD (*r* = −0.27, *p* = 0.123). Mortality was more prevalent among subjects with IPAQ ≤ 417 METS-min/week and ∆SpO_2_ ≥ 10% (Table 3).

## 4. Discussion

This prospective follow-up study sought to evaluate the prognostic utility of PA and ED among IPF patients. We found that PA levels ≤417 METS-min/week and ∆SpO_2_ ≥ 10% in 6MWT were significantly associated with mortality in patients with IPF (Table 2 and Table 3; Figure 1 and Figure 2). In this regard, we have previously demonstrated that exercise training intervention did not show survival benefits at 30 [18] and at 40 [19] months follow-up, although the sample size was underpowered to detect such differences. Our previous report also showed several additional variables using pulmonary and ventilatory functions as well as exercise capacity in association with mortality among IPF patients [19]. In the current study, we found significant correlation (*r* = 0.57, *p* < 0.001) between PA and 6MWD, which strengthens the findings, since the latter is a well-established prognostic marker in IPF [6,37,38,39]. These data emphasize the utility and the valuable role of PA and the level of ED evaluation for risk stratification and prognosis in IPF. The findings also underscore the possible survival benefits of even low levels of PA (>417 METS-min/week corresponding to ~100–105 min/week of moderate intensity PA) for patients with IPF. Simple assessment (5 to 10 min interview) of PA levels using IPAQ and ED in 6MWT, as we described here, can be easily implemented and used as a good practical aid for clinical decision making and treatment, patients’ consultation for PA, and for prioritizing referral for pulmonary rehabilitation and lung transplantation. Physical inactivity seems to be an important modifiable risk factor for mortality among IPF that should be addressed more deeply with future research.

The results of the current study provide novel evidence on the importance of PA and ED for survival in IPF. These findings are consistent with a previous large cohort study showing mortality risk reduction with only 15 min of daily PA in a general population [40]. Our findings also support previous studies showing that inactivity was associated with increased risk of mortality among subjects with ILD [6,12]. Wallaert et al. [6] demonstrated a significant cut-off point of 3287 steps/day with hazard ratio of 2.72 for mortality among fibrotic idiopathic interstitial pneumonia patients [6]. This threshold is considered well-below the general PA recommendation for elderly and chronic disease patients (i.e., 7000–8000 steps/day) [41], and aligns with our determined cut-off point (417 METS-min/week) of PA, which was also lower than the general recommended PA levels (500–1000 METS-min/week) [25]. Leuchte et al. [12] also found a poorer survival with 16.9-fold increased mortality risk in IPF patients who were classified as low active based on self-report PA [12]. These results align with our findings of 9.7-fold increased mortality risk in patients who reported PA below 417 METS-min/week. Furthermore, our data corroborate previous studies showing poorer survival rates in IPF patients with significant desaturation in 6MWT [4,42]. The present study’s advantages are addressing some of the clinical unresolved gaps, adopting a prospective follow-up, using sensitive thresholds for variables, reporting both the levels of sensitivity and specificity, and indicating the hazard ratios for death using novel predictors (Table 2 and Table 3). Previous studies found a threshold of SpO_2_ < 88% during 6MWT as a strong prognostic marker in IPF [4,42]. However, this absolute variable may be less sensitive for prognostication of patients who already experience some degree of resting hypoxemia. Exercise-induced desaturation might be different for various resting SpO_2_ levels. In this regard, desaturation of 10% or more during 6MWT was significantly associated with mortality, which overcomes the variation in resting SpO_2_ levels across patients. Furthermore, the findings demonstrated for the first time in an IPF group that PA levels assessed by IPAQ are associated with mortality, and even low activity levels (i.e., below the general PA recommendation) revealed a significant reduced risk for death [7,43,44]. These findings are consistent with data reported in COPD patients on the survival benefits of even low PA levels [45,46]. Moreover, the results presented here provide novel insights to a new and easily implemented assessment of practical prognostic predictors of mortality (IPAQ ≤ 417 METS-min/week and ∆SpO_2_ ≥ 10%) that can enhance the overall risk stratification and add important information in the process of decision making and treatment options for patients with IPF, which further studies using large sample sizes should explore more extensively. 

Possible mechanistic explanations for these findings can be related to the fact that physical inactivity is a well-established all-cause cardiovascular and respiratory mortality risk factor [7,13,14,46,47,48,49]. Regular engagement in PA was well documented in reducing the incidence of many chronic conditions, such as coronary artery disease, type-2 diabetes, stroke, several types of cancers, obesity, depression, and falls in elderliness. Moreover, the American College of Sports Medicine stated that PA is an effective evidence-based strategy to improve cardiorespiratory, metabolic, and musculoskeletal health, enhance well-being, as well as limit the progression of chronic diseases and disability in older adults [44]. Recently, ATS/ERS position stand (2013) documented that inactivity among chronic respiratory disease patients is associated with poorer outcomes, including higher mortality risk [10]. These reports were confirmed with findings of inactivity associated with poorer survival in patients with IPF [6,12]. It is possible that even low PA levels (below the general PA recommendation of 150 min/week of moderate intensity)—as this study and others demonstrated—have a protective effect against disease progression and mortality, especially in ill and elderly populations, as in IPF patients [6,12,25]. This important issue needs to be ascertained in future large prospective studies.

ED was also clearly demonstrated as a significant risk factor for death and was associated with more severe conditions of IPF disease [1,4]. Higher levels of desaturation during exercise usually suggests extended parenchymal fibrosis and an advanced disease stage, pronounced gas exchange abnormalities, severe dyspnea, and worsened survival in IPF [1,34,50,51]. Our findings showed that ∆SpO_2_ ≥ 10% is a good sensitive threshold for detecting high risk IPF patients, and as such may be considered for implementation in clinical and research settings, due to the low cost, ease of assessment, and practical availability. However, despite the strong association between PA and ED with mortality observed in the current study, given the multifactorial manifestation of the disease and the diversity of predictors, we believe that prognosis should be based on a combination of predictors rather than on a single variable, and future trials should address this issue using large cohorts and multivariate analyses.

A few possible limitations in our study are related to the small sample size and the level of IPF severity among patients (median: forced vital capacity (FVC); 68% predicted), thus caution should be taken with generalization of our findings. Despite these limitations, the study has a number of strengths: collecting data prospectively, analyses using various statistical methods for the detection of sensitive thresholds, and calculating the risk for mortality and survival analysis. Moreover, the study found novel, sensitive, and practical indicators that can be easily implemented in clinical practice. The number of participants in our cohort is comparable to a previous study of PA and prognosis in IPF [6]. An additional strength of the study is that PA levels were reported using one-on-one personalized interviews rather than self-filling questionnaires, which probably increased the accuracy of the self-reported PA. Moreover, IPAQ is accepted as a reliable and validated tool for the assessment of PA in many research and clinical settings [22,23,24,52], and recently was also studied in IPF subjects [53,54].

In summary, this prospective follow-up study originates new, simple, and practical prognostic cut-off points of PA levels and ED in IPF subjects. The results demonstrated that PA levels ≤417 METS-min/week and ∆SpO_2_ ≥ 10% in 6MWT were significantly associated with increased risk of mortality. This study strengthens the evidence for assessing PA and ED for the detection of high-risk IPF patients and aids in addressing appropriate treatments, such as PA consultation, pulmonary rehabilitation programs, oxygen supplementation, and referral for lung transplantation. The findings also underscore that even low levels of PA seem to have prognostic benefits for IPF patients. We suggest considering physical inactivity as a new independent modifiable prognostic risk factor for IPF. Further research with large cohorts is warranted to encourage implementation and the establishment of PA assessments in standard clinical practice.

## Figures and Tables

**Table 1:** Baseline Demographic Characteristics and Variables of Study Population (*n* = 34).

Variables	Values
Age (years)	68 (50–81)
Male/Female (*n*/%)	22/12 (65%/35%)
Body mass index	29 (22–37)
Time from diagnosis (years)	1 (0.1–15)
Patients with smoking history (*n*/%)	20 (59%)
Packs/year	27 (0–112)
**Supplemental oxygen users**	
Rest	4 (12%)
Exertion	9 (26%)
**Co-morbidities**	
Pulmonary hypertension according to echocardiography (*n*/%)	13 (38%)
Coronary arterial disease (*n*/%)	14 (41%)
Systemic hypertension (*n*/%)	24 (71%)
Chronic obstructive pulmonary disease–Emphysema (*n*/%)	8 (24%)
Type 2 Diabetes (*n*/%)	13 (38%)
Osteoporosis (*n*/%)	5 (15%)
**Medications**	
Corticosteroids (*n*/%)	23 (68%)
Pirfenidone (*n*/%)	3 (9%)
Beta blockers (*n*/%)	13 (38%)
**Modified Medical Research Council-dyspnea scale (0–4)**	
0	1 (3%)
1	14 (41%)
2	8 (24%)
3	10 (29%)
4	1 (3%)
**Resting cardiopulmonary parameters**	
FVC % predicted	68 (37–109)
FEV_1_ % predicted	70 (35–124)
TLC % predicted	64 (35–99)
DLCO % predicted	50 (23–91)
SpO_2_ at rest (%)	97 (87–99)
**Exercise capacity, physical activity, and desaturation**	
6MWD (m)	505 (130–749)
% predicted	99 (35–147)
IPAQ (METS-min/week)	648 (0–12,200)
∆SpO_2_ (%)	9 (0–27)

Values are presented as median (range). FVC: forced vital capacity; FEV_1_: forced expiratory volume i; DLCO: diffusion capacity for carbon monoxide; TLC: total lung capacity; SpO_2_: oxygen saturation by pulse oximeter; 6MWD: 6 min walking distance; ∆SpO_2_: oxygen saturation by pulse oximeter pre-to-post 6 min walking test.

**Table 2:** Cut-off points of physical activity and exertional desaturation predicting mortality in patients with idiopathic pulmonary fibrosis.

Cut-off Point for Parameters	AUC CI (95%)	Sensitivity (%)	Specificity (%)	*p*-Value
IPAQ ≤ 417 (MET-min/week)	0.808 (0.66–0.96)	82	70	0.004
∆SpO_2_ ≥ 10 (%)	0.785 (0.28–0.77)	91	74	0.008

AUC: area under the curve; CI: confidence interval; IPAQ: international physical activity questionnaire; MET: metabolic equivalent task; ∆SpO_2_: difference of oxygen saturation pre- to post-6 min walking test measured by pulse oximeter.

**Table 3:** Physical activity and exertional desaturation characteristics in survivor and non-survivor idiopathic pulmonary fibrosis patients.

	Survivors	Non-Survivors	Total	*p*-Value
IPAQ > 417 (MET-min/week)	18 (90%)	2 (10%)	20 (100%)	0.001
IPAQ ≤ 417 (MET-min/week)	5 (35.7%)	9 (64.3%)	14 (100%)	
∆SpO_2_ < 10 (%)	17 (94.4%)	1 (5.6%)	18 (100%)	<0.001
∆SpO_2_ ≥ 10 (%)	6 (37.5%)	10 (62.5%)	16 (100%)	

Data presented as absolute numbers and (percent of the group). *p* was calculated using Chi Square test for categorical variables. IPAQ: international physical activity questionnaire; ∆SpO_2_: difference of oxygen saturation pre to post 6 min walking test measured by pulse oximeter.

## References

[1] Raghu G., Collard H.R., Egan J.J., Martinez F.J., Behr J., Brown K.K., Colby T.V., Cordier J.F., Flaherty K.R., Lasky J.A. (2011). An official ATS/ERS/JRS/ALAT statement: Idiopathic pulmonary fibrosis: Evidence-based guidelines for diagnosis and management. Am. J. Respir. Crit. Care Med..

[2] Olson A.L., Swigris J.J., Lezotte D.C., Norris J.M., Wilson C.G., Brown K.K. (2007). Mortality from pulmonary fibrosis increased in the United States from 1992 to 2003. Am. J. Respir. Crit. Care Med..

[3] Meltzer E.B., Noble P.W. (2008). Idiopathic pulmonary fibrosis. Orphanet J. Rare Dis..

[4] Lama V.N., Flaherty K.R., Toews G.B., Colby T.V., Travis W.D., Long Q., Murray S., Kazerooni E.A., Gross B.H., Lynch J.P. (2003). Prognostic value of desaturation during a 6-min walk test in idiopathic interstitial pneumonia. Am. J. Respir. Crit. Care Med..

[5] Swigris J.J., Brown K.K., Make B.J., Wamboldt F.S. (2008). Pulmonary rehabilitation in idiopathic pulmonary fibrosis: A call for continued investigation. Respir. Med..

[6] Wallaert B., Monge E., Le Rouzic O., Wemeau-Stervinou L., Salleron J., Grosbois J.M. (2013). Physical activity in daily life of patients with fibrotic idiopathic interstitial pneumonia. Chest.

[7] World Health Organization (2010). Global Recommendations on Physical Activity for Health.

[8] Hallal P.C., Andersen L.B., Bull F.C., Guthold R., Haskell W., Ekelund U., Lancet Physical Activity Series Working Group (2012). Global physical activity levels: Surveillance progress, pitfalls, and prospects. Lancet.

[9] Lee I.M., Shiroma E.J., Lobelo F., Puska P., Blair S.N., Katzmarzyk P.T., Lancet Physical Activity Series Working Group (2012). Effect of physical inactivity on major non-communicable diseases worldwide: An analysis of burden of disease and life expectancy. Lancet.

[10] Spruit M.A., Singh S.J., Garvey C., Zuwallack R., Nici L., Rochester C., Hill K., Holland A.E., Lareau S.C., Man W.D. (2013). An official american thoracic society/european respiratory society statement: Key concepts and advances in pulmonary rehabilitation. Am. J. Respir. Crit. Care Med..

[11] Nakayama M., Bando M., Araki K., Sekine T., Kurosaki F., Sawata T., Nakazawa S., Mato N., Yamasawa H., Sugiyama Y. (2015). Physical activity in patients with idiopathic pulmonary fibrosis. Respirology.

[12] Leuchte H.H., Mernitz P., Baezner C., Baumgartner R.A., von Wulffen W., Neurohr C., Behr J. (2015). Self-Report Daily Life Activity as a Prognostic Marker of Idiopathic Pulmonary Fibrosis. Respiration.

[13] Bouchard C., Blair S.N., Katzmarzyk P.T. (2015). Less Sitting, More Physical Activity, or Higher Fitness?. Mayo Clin. Proc..

[14] Watz H., Pitta F., Rochester C.L., Garcia-Aymerich J., ZuWallack R., Troosters T., Vaes A.W., Puhan M.A., Jehn M., Polkey M.I. (2014). An official European Respiratory Society statement on physical activity in COPD. Eur. Respir. J..

[15] Vainshelboim B., Oliveira J., Yehoshua L., Weiss I., Fox B.D., Fruchter O., Kramer M.R. (2014). Exercise Training-Based Pulmonary Rehabilitation Program Is Clinically Beneficial for Idiopathic Pulmonary Fibrosis. Respiration.

[16] Florey C.D. (1993). Sample size for beginners. BMJ.

[17] Holland A.E., Hill C.J., Conron M., Munro P., McDonald C.F. (2008). Short term improvement in exercise capacity and symptoms following exercise training in interstitial lung disease. Thorax.

[18] Vainshelboim B., Oliveira J., Fox B.D., Soreck Y., Fruchter O., Kramer M.R. (2015). Long-term effects of a 12-week exercise training program on clinical outcomes in idiopathic pulmonary fibrosis. Lung.

[19] Vainshelboim B., Oliveira J., Fox B.D., Kramer M.R. (2016). The Prognostic Role of Ventilatory Inefficiency and Exercise Capacity in Idiopathic Pulmonary Fibrosis. Respir. Care.

[20] Mahler D.A., Wells C.K. (1988). Evaluation of clinical methods for rating dyspnea. Chest.

[21] Papiris S.A., Daniil Z.D., Malagari K., Kapotsis G.E., Sotiropoulou C., Milic-Emili J., Roussos C. (2005). The Medical Research Council dyspnea scale in the estimation of disease severity in idiopathic pulmonary fibrosis. Respir. Med..

[22] Craig C.L., Marshall A.L., Sjostrom M., Bauman A.E., Booth M.L., Ainsworth B.E., Pratt M., Ekelund U., Yngve A., Sallis J.F. (2003). International physical activity questionnaire: 12-Country reliability and validity. Med. Sci. Sports Exerc..

[23] Booth M. (2000). Assessment of physical activity: An international perspective. Res. Q. Exerc. Sport.

[24] Lee P.H., Macfarlane D.J., Lam T.H., Stewart S.M. (2011). Validity of the International Physical Activity Questionnaire Short Form (IPAQ-SF): A systematic review. Int. J. Behav. Nutr. Phys. Act..

[25] Garber C.E., Blissmer B., Deschenes M.R., Franklin B.A., Lamonte M.J., Lee I.M., Nieman D.C., Swain D.P., American College of Sports Medicine (2011). American College of Sports Medicine position stand. Quantity and quality of exercise for developing and maintaining cardiorespiratory, musculoskeletal, and neuromotor fitness in apparently healthy adults: Guidance for prescribing exercise. Med. Sci. Sports Exerc..

[26] Pescatello L.S., American College of Sports Medicine (2014). ACSM’s Guidelines for Exercise Testing and Prescription.

[27] Haskell W.L., Lee I.M., Pate R.R., Powell K.E., Blair S.N., Franklin B.A., Macera C.A., Heath G.W., Thompson P.D., Bauman A. (2007). Physical activity and public health: Updated recommendation for adults from the American College of Sports Medicine and the American Heart Association. Circulation.

[28] Pate R.R., Pratt M., Blair S.N., Haskell W.L., Macera C.A., Bouchard C., Buchner D., Ettinger W., Heath G.W., King A.C. (1995). Physical activity and public health. A recommendation from the Centers for Disease Control and Prevention and the American College of Sports Medicine. JAMA.

[29] ATS Committee on Proficiency Standards for Clinical Pulmonary Function Laboratories (2002). ATS statement: Guidelines for the six-minute walk test. Am. J. Respir. Crit. Care Med..

[30] Macintyre N., Crapo R.O., Viegi G., Johnson D.C., van der Grinten C.P., Brusasco V., Burgos F., Casaburi R., Coates A., Enright P. (2005). Standardisation of the single-breath determination of carbon monoxide uptake in the lung. Eur. Respir. J..

[31] Miller M.R., Crapo R., Hankinson J., Brusasco V., Burgos F., Casaburi R., Coates A., Enright P., van der Grinten C.P., Gustafsson P. (2005). General considerations for lung function testing. Eur. Respir. J..

[32] Wanger J., Clausen J.L., Coates A., Pedersen O.F., Brusasco V., Burgos F., Casaburi R., Crapo R., Enright P., van der Grinten C.P. (2005). Standardisation of the measurement of lung volumes. Eur. Respir. J..

[33] Quanjer P.H., Tammeling G.J., Cotes J.E., Pedersen O.F., Peslin R., Yernault J.C. (1993). Lung volumes and forced ventilatory flows. Report Working Party Standardization of Lung Function Tests, European Community for Steel and Coal. Official Statement of the European Respiratory Society. Eur. Respir. J. Suppl..

[34] Mura M., Porretta M.A., Bargagli E., Sergiacomi G., Zompatori M., Sverzellati N., Taglieri A., Mezzasalma F., Rottoli P., Saltini C. (2012). Predicting survival in newly diagnosed idiopathic pulmonary fibrosis: A 3-year prospective study. Eur. Respir. J..

[35] Wallaert B., Guetta A., Wemeau-Stervinou L., Terce G., Valette M., Neviere R., Aguilaniu B. (2011). [Prognostic value of clinical exercise testing in idiopathic pulmonary fibrosis]. Rev. Mal. Respir..

[36] Mukaka M.M. (2012). Statistics corner: A guide to appropriate use of correlation coefficient in medical research. Malawi Med. J..

[37] du Bois R.M., Albera C., Bradford W.Z., Costabel U., Leff J.A., Noble P.W., Sahn S.A., Valeyre D., Weycker D., King T.E. (2014). 6-Min walk distance is an independent predictor of mortality in patients with idiopathic pulmonary fibrosis. Eur. Respir. J..

[38] Caminati A., Bianchi A., Cassandro R., Mirenda M.R., Harari S. (2009). Walking distance on 6-MWT is a prognostic factor in idiopathic pulmonary fibrosis. Respir. Med..

[39] Lederer D.J., Arcasoy S.M., Wilt J.S., D’Ovidio F., Sonett J.R., Kawut S.M. (2006). Six-minute-walk distance predicts waiting list survival in idiopathic pulmonary fibrosis. Am. J. Respir. Crit. Care Med..

[40] Lee D.C., Pate R.R., Lavie C.J., Sui X., Church T.S., Blair S.N. (2014). Leisure-time running reduces all-cause and cardiovascular mortality risk. J. Am. Coll. Cardiol..

[41] Tudor-Locke C., Craig C.L., Aoyagi Y., Bell R.C., Croteau K.A., De Bourdeaudhuij I., Ewald B., Gardner A.W., Hatano Y., Lutes L.D. (2011). How many steps/day are enough? For older adults and special populations. Int. J. Behav. Nutr. Phys. Act..

[42] Flaherty K.R., Andrei A.C., Murray S., Fraley C., Colby T.V., Travis W.D., Lama V., Kazerooni E.A., Gross B.H., Toews G.B. (2006). Idiopathic pulmonary fibrosis: Prognostic value of changes in physiology and six-minute-walk test. Am. J. Respir. Crit. Care Med..

[43] Nelson M.E., Rejeski W.J., Blair S.N., Duncan P.W., Judge J.O., King A.C., Macera C.A., Castaneda-Sceppa C. (2007). Physical activity and public health in older adults: Recommendation from the American College of Sports Medicine and the American Heart Association. Circulation.

[44] Chodzko-Zajko W.J., Proctor D.N., Fiatarone Singh M.A., Minson C.T., Nigg C.R., Salem G.J., Skinner J.S. (2009). American College of Sports Medicine position stand. Exercise and physical activity for older adults. Med. Sci. Sports Exerc..

[45] Garcia-Aymerich J., Lange P., Benet M., Schnohr P., Anto J.M. (2006). Regular physical activity reduces hospital admission and mortality in chronic obstructive pulmonary disease: A population based cohort study. Thorax.

[46] Waschki B., Kirsten A., Holz O., Muller K.C., Meyer T., Watz H., Magnussen H. (2011). Physical activity is the strongest predictor of all-cause mortality in patients with COPD: A prospective cohort study. Chest.

[47] Hupin D., Roche F., Gremeaux V., Chatard J.C., Oriol M., Gaspoz J.M., Barthelemy J.C., Edouard P. (2015). Even a low-dose of moderate-to-vigorous physical activity reduces mortality by 22% in adults aged ≥60 years: A systematic review and meta-analysis. Br. J. Sports Med..

[48] Warburton D.E., Nicol C.W., Bredin S.S. (2006). Health benefits of physical activity: The evidence. Can. Med. Assoc. J..

[49] Wen C.P., Wai J.P., Tsai M.K., Yang Y.C., Cheng T.Y., Lee M.C., Chan H.T., Tsao C.K., Tsai S.P., Wu X. (2011). Minimum amount of physical activity for reduced mortality and extended life expectancy: A prospective cohort study. Lancet.

[50] Vainshelboim B., Oliveira J., Fox B.D., Adir Y., Ollech J.E., Kramer M.R. (2016). Physiological Profile and Limitations in Exercise in Idiopathic Pulmonary Fibrosis. J. Cardiopulm. Rehabil. Prev..

[51] Lee S.H., Shim H.S., Cho S.H., Kim S.Y., Lee S.K., Son J.Y., Jung J.Y., Kim E.Y., Lim J.E., Lee K.J. (2011). Prognostic factors for idiopathic pulmonary fibrosis: Clinical, physiologic, pathologic, and molecular aspects. Sarcoidosis Vasc. Diffuse Lung Dis..

[52] Helmerhorst H.J., Brage S., Warren J., Besson H., Ekelund U. (2012). A systematic review of reliability and objective criterion-related validity of physical activity questionnaires. Int. J. Behav. Nutr. Phys. Act..

[53] Gaunaurd I.A., Gomez-Marin O.W., Ramos C.F., Sol C.M., Cohen M.I., Cahalin L.P., Cardenas D.D., Jackson R.M. (2014). Physical activity and quality of life improvements of patients with idiopathic pulmonary fibrosis completing a pulmonary rehabilitation program. Respir. Care.

[54] Vainshelboim B., Fox B.D., Kramer M.R., Izhakian S., Gershman E., Oliveira J. (2016). Short-Term Improvement in Physical Activity and Body Composition After Supervised Exercise Training Program in Idiopathic Pulmonary Fibrosis. Arch. Phys. Med. Rehabil..

